# Frequent CXCR4 tropism of HIV-1 subtype A and CRF02_AG during late-stage disease - indication of an evolving epidemic in West Africa

**DOI:** 10.1186/1742-4690-7-23

**Published:** 2010-03-22

**Authors:** Joakim Esbjörnsson, Fredrik Månsson, Wilma Martínez-Arias, Elzbieta Vincic, Antonio J Biague, Zacarias J da Silva, Eva Maria Fenyö, Hans Norrgren, Patrik Medstrand

**Affiliations:** 1 Department of Experimental Medical Science, Section of Molecular Virology, Lund University, Lund, Sweden; 2 Department of Clinical Sciences, Malmö, Infectious Diseases Research Unit, Lund University, Sweden; 3 Department of Laboratory Medicine Lund, Section of Virology, Lund University, Lund, Sweden; 4 Department of Clinical Sciences, Division of Infection Medicine, Lund University, Lund, Sweden; 5 National Public Health Laboratory, Bissau, Guinea-Bissau

## Abstract

**Background:**

HIV-1 is one of the fastest evolving pathogens, and is distinguished by geographic and genetic variants that have been classified into different subtypes and circulating recombinant forms (CRFs). Early in infection the primary coreceptor is CCR5, but during disease course CXCR4-using HIV-1 populations may emerge. This has been correlated with accelerated disease progression in HIV-1 subtype B. Basic knowledge of HIV-1 coreceptor tropism is important due to the recent introduction of coreceptor antagonists in antiretroviral therapy, and subtype-specific differences regarding how frequently HIV-1 CXCR4-using populations appear in late-stage disease need to be further investigated. To study how frequently CXCR4-using populations appear in late-stage disease among HIV-1 subtype A and CRF02_AG, we evaluated the accuracy of a recombinant virus phenotypic assay for these subtypes, and used it to determine the HIV-1 coreceptor tropism of plasma samples collected during late-stage disease in Guinea-Bissau. We also performed a genotypic analysis and investigated subtype-specific differences in the appearance of CXCR4 tropism late in disease.

**Results:**

We found that the recombinant virus phenotypic assay accurately predicted HIV-1 coreceptor tropism of subtype A and CRF02_AG. Over the study period (1997-2007), we found an increasing and generally high frequency of CXCR4 tropism (86%) in CRF02_AG. By sequence analysis of the V3 region of our samples we developed a novel genotypic rule for predicting CXCR4 tropism in CRF02_AG, based on the combined criteria of the total number of charged amino acids and net charge. This rule had higher sensitivity than previously described genotypic rules and may be useful for development of future genotypic tools for this CRF. Finally, we conducted a literature analysis, combining data of 498 individuals in late-stage disease, and found high amounts of CXCR4 tropism for all major HIV-1 subtypes (60-77%), except for subtype C (15%).

**Conclusions:**

The increase in CXCR4 tropism over time suggests an evolving epidemic of CRF02_AG. The results of the literature analysis demonstrate the need for further studies investigating subtype-specific emergence for CXCR4-tropism; this may be particularly important due to the introduction of CCR5-antagonists in HIV treatment regimens.

## Background

Human immunodeficiency virus type 1 (HIV-1) evolves at an extremely high rate, primarily due to a combination of high viral turn-over, an error prone viral reverse transcriptase and frequent recombination. This high level of molecular evolution has led to diversification of HIV-1 into genetically distinct subtypes (A-D, F-H, J-K), subsubtypes (A1-A3, F1-F2) and circulating recombinants forms (CRFs), usually defined by geographical location [1]. The most common subtypes are subtype A (12.3% of the global prevalence), B (10.2%), C (49.9%) and G (6.3%), and the CRF01_AE (4.7%) and CRF02_AG (4.8%) [1].

HIV-1 enters target cells via interactions with CD4 and a coreceptor, usually one of the chemokine receptors CCR5 or CXCR4. Different HIV strains have been classified based on coreceptor tropism: CCR5-tropic strains are referred to as R5, CXCR4-tropic strains as X4, and dual tropic strains as R5X4 [2]. Coreceptor use has been studied extensively in HIV-1 subtype B and C, but needs further investigation for other subtypes [3-13]. In subtype B, R5 populations are generally present over the entire course of infection whereas R5X4 or X4 populations emerge late in infection. This coreceptor switch has been associated with faster CD4+ T cell decline and the development of AIDS, although studies describing the opposite, or no difference in CD4+ T cell decline have also been observed [5,6].

Little is known about subtype-specific differences regarding how frequently CXCR4-using populations appear in late-stage disease. Most studies investigating HIV-1 subtype B coreceptor tropism have focused on either the relation between the detection of X4 viruses and disease progression rate, or molecular properties that differ between the R5 and X4 viruses [5,6,14,15]. HIV-1 CXCR4-using populations are thought to appear in approximately 50% of the patients infected with subtype B [16-18]. The fraction of subtype C-infected individuals that have CXCR4-using populations appear to be less frequent (0-30%) [8,9,11,19]. Moreover, a study of HIV-1 CRF01_AE in 22 AIDS patients showed that 16 subjects (73%) had X4 populations [12]. Comparing the A and D subtypes, Kaleebu et al. found no significant difference in patients with low CD4 counts (≤ 200) [3]. When a comparison was done at an earlier stage of HIV-1 infection (CD4 counts > 200), CXCR4 use was more frequent among patients with subtype D infection, probably due to an earlier coreceptor switch than in patients infected with subtype A [3]. Other cross-sectional studies do not allow estimation of the emergence of HIV-1 with X4 phenotype since CD4 counts or clinical statuses were not considered together [20-22].

The viral envelope glycoprotein (gp) 120 is organized in five hypervariable regions (V1-V5), interspersed within five conserved regions (C1-C5). The major viral determinants of the interaction between gp120 and the coreceptors CCR5 or CXCR4 are located in the V3 region, even though other regions, such as the V1/V2 and the C4 regions have been shown to influence coreceptor use [23,24]. To date, most studies have focused on HIV-1 subtype B and C, and there is no clear evidence that the V3 region has the same impact on coreceptor interaction among other subtypes.

Basic knowledge of HIV-1 coreceptor evolution has become increasingly important due to the recent introduction of CCR5 antagonists as part of antiretroviral therapy against HIV-1 [25,26]. Since these drugs have no effect on X4 populations, HIV-1 coreceptor tropism must be identified before the initiation of treatment [27,28]. The gold standard for clinical samples is coreceptor determination by recombinant phenotypic entry assays [29,30]. Reliable bioinformatic tools based on viral genotype may be a faster and more cost-effective way to predict coreceptor tropism. Present genotypic predictors are based on V3 sequences from subtype B or C, and have been shown to perform poorly on other subtypes, especially in detecting CXCR4-using variants [31,32]. This indicates that molecular differences connected to coreceptor use may be subtype-specific, and that specific predictors likely have to be constructed for each major subtype and CRF.

In view of this, we set out to determine the frequency of emergence of X4 phenotype in HIV-1 subtype A or CRF02_AG infected individuals in late-stage disease by evaluating the performance of a recombinant virus phenotypic assay for subtype A and CRF02_AG. Using this tool, we found an increasing and generally high frequency of CXCR4 tropism (86%) in CRF02_AG, and developed a novel genotypic rule for predicting CXCR4 tropism, based on combined criteria of the total number of charged amino acids and net charge of the V3 region. Finally, we compared our results to other HIV-1 subtypes by analyzing HIV-1 coreceptor phenotype in individuals in late-stage disease and found high amounts of CXCR4 tropism for all major HIV-1 subtypes (60-77%), except for subtype C (15%).

## Methods

### Sample sets

Two sample sets were used in the present study. The first sample set consisted of a control panel of 11 HIV-1 isolates with predetermined subtype and coreceptor tropism. Subtype was determined by sequencing of the *env *V3 region, and coreceptor tropism was determined using a phenotypic infection assay with either the U87.CD4-CCR5/U87.CD4-CXCR4 cell system or the MT-2 cell system [22]. Data were generously provided by Professor Jan Albert, Swedish Institute for Infectious Disease Control, Stockholm, Sweden. All isolates were amplified, sequenced, and used for evaluation of the recombinant virus phenotypic assay. Three isolates (22480, 22627, and 30405) were also used in the genotypic analysis of HIV-1 CRF02_AG. Details of the control panel can be found in Table 1. The second sample set consisted of 33 plasma samples from 33 HIV-1 infected individuals and was selected from a cohort of police officers from Guinea-Bissau, West Africa, based on sample availability and disease status. The cohort has been described in detail elsewhere [33-35]. Twenty-nine of the samples were successfully amplified and subjected to further analyses. All of the individuals were treatment naïve and classified to be in late-stage disease, as defined by CD4+ T cell count (≤ 200 cells/μl or ≤ 14%) or clinical AIDS (CDC: C or WHO: 4) [36,37]. In cases where more than one sample from late-stage disease was available, the last sample was chosen. Individuals diagnosed with tuberculosis and clinically categorized as CDC: C, but without other AIDS-defining symptoms, were not included in the study. For patient samples DL2713H, DL2846I and DL3018H, there were no recorded CD4+ T cell counts. These samples were included in the study based on previous observations of CD4+ T cell counts of the same patient, according to the described criterions. Details of the plasma samples from Guinea-Bissau can be found in Table 2.

**Table 1:** Evaluation of the TRT assay using a control panel of HIV-1 subtype A and CRF02_AG isolates.

	Coreceptor tropism		
Isolate No.			Subtype
	Isolate	Chimeric virus^1^	
7535	R5	R5	A
9488	R5	R5	A
22480	R5	R5	CRF02_AG
22627	R5	R5	CRF02_AG
36412	R5	R5	A
36748	R5	R5	A
8131	R5X4	R5X4	A
30405	R5X4	R5X4	CRF02_AG
9284	R5X4	R5X4	A
11974	X4	R5X4	A
13636	R5X4	R5X4	A

^1^Chimeric viruses were constructed using amplified HIV-1 gp120 V1-V3 fragments specific for each isolate according to the protocol of the TRT assay.

**Table 2:** Clinical parameters, HIV-1 subtype and HIV-1 tropism of the 29 analyzed study subjects.

Patient No.	Sex^1^	CD4%^2^	CD4tot^3^	CDC^4^	WHO^5^	Subtype	Tropism	Sample year
DL1996H	M	5	157	B	3	CRF02_AG	R5X4	2000
DL2089J	M	9	59	B	3	CRF02_AG	R5X4	2003
DL2096F	M	5	22	C	4	C	N/A^6^	2003
DL2249I	M	2	21	C	4	CRF02_AG	R5X4	2004
DL2339E	M	12	178	B	3	CRF02_AG	R5X4	2003
DL2365K	M	9	133	B	3	A3	R5	2006
DL2391G	M	5	N/A^6^	B	2	CRF02_AG	R5	2000
DL2401M	M	11	141	B	3	CRF02_AG	R5X4	2004
DL2713H	M	N/A^6^	N/A^6^	B	2	CRF02_AG	R5X4	2007
DL2846I	F	N/A^6^	N/A^6^	B	3	A3	R5X4	2005
DL2853E	M	11	137	A	1	CRF02_AG	R5	1998
DL2920H	M	11	126	B	3	CRF02_AG	X4	2004
DL3018H	M	N/A^6^	N/A^6^	B	3	A3	R5X4	2006
DL3037E	M	3	74	B	3	CRF02_AG	R5X4	2005
DL3039G	F	7	148	A	2	CRF02_AG	R5X4	2006
DL3071H	F	20	123	B	3	A3	R5X4	2005
DL3087E	M	4	62	B	2	CRF02_AG	R5X4	2001
DL3098I	F	14	426	N/A^6^	N/A^6^	CRF02_AG	R5X4	2007
DL3169F	M	9	315	B	3	CRF02_AG	R5X4	2004
DL3170F	M	8	65	B	2	CRF02_AG	R5X4	2000
DL3234J	M	10	216	A	2	CRF02_AG	R5X4	2006
DL3312E	M	2	36	C	4	CRF02_AG	R5X4	1998
DL3633G	F	8	112	C	4	CRF02_AG	R5X4	2003
DL3721C	M	11	257	A	1	CRF02_AG	R5X4	1997
DL3733G	M	19	137	B	3	CRF02_AG	R5X4	2004
DL4248G	F	13	159	B	3	A3	R5	2005
DL4477D	M	14	141	B	3	CRF02_AG	R5	2001
DL4525G	M	13	372	B	3	A3	R5	2006
DL4632E	F	9	77	B	3	CRF02_AG	R5X4	2003

^1^M = male, F = female

^2^CD4+ T cell percentage among all T cells

^3^CD4+ T cell count per microliter among all T cells

^4^Clinical category of the patient, as defined by the CDC, at the sample time point

^5^Clinical category of the patient, as defined by the WHO, at the sample time point

^6^N/A = not analyzed

^7^Sample included in the study based on previous examinations of CD4+ T cell count and percentage

### Amplification and sequencing

Viral RNA was extracted and purified from blood plasma samples, using RNeasy Lipid Tissue Mini Kit (Qiagen, Stockholm, Sweden) with minor modifications from the manufacturer's instructions. Briefly, 200 μl of blood plasma were disrupted in 2000 μl Qiazol and 10 μg Carrier RNA (Qiagen). The aqueous phase was loaded onto a spin column by multiple loading steps. RNA was eluted in 40 μl of RNase-free water and treated with DNase I (Fermentas, Helsingborg, Sweden). Viral RNA was reverse transcribed using gene-specific primers, and the V1-V3 region amplified using a nested PCR approach (The SuperScript™ III One-Step RT-PCR System with Platinum^® ^*Taq *DNA Polymerase and Platinum^® ^*Taq *DNA Polymerase High Fidelity, Invitrogen, Copenhagen, Denmark) according to the manufacturer's instructions using primers JE12F (5'-AAAGAGCAGAAGATAGTGGCAATGA-3') and V3A_R2 (5'-TTACAATAGAAAAATTCTCCTCYACA-3') for one-step RT-PCR and E20A_F (5'-GGGCTACACATGCCTGTGTACCYACAG-3') and JA169 for nested PCR [38]. The V3 region with flanking regions (nucleotides 6847 to 7374 in HXB2; GenBank accession number K03455) were then directly sequenced using BigDye Terminator v1.1 Cycle Sequencing Kit (Applied Biosystems, Stockholm, Sweden) according to the manufacturer's instructions using primers JA167 and JA169 [38]. The V3 region was chosen for sequencing due to prevalence of length variations in the V1-V2 region. Sequences were determined using ABI Prism 3100 (Applied Biosystems). Sample DL2846I had to be cloned as a result of too mixed chromatograms. The amplified V1-V3 region of approximately 940 base pairs (nucleotides 6430 to 7374 in HXB2; GenBank accession number K03455) were cloned using the InsTAclone cloning system (Fermentas) and TOP10 cells (Invitrogen). Twelve colonies were picked and the cloned fragments were amplified with Platinum^® ^*Taq *DNA Polymerase High Fidelity (Invitrogen) using conventional M13 primers (-20 and -24). The amplifications were successful for eight colonies, and clones were named with the patient identification number and a clone number. Individual clones were purified and sequenced as described.

### Phylogenetic analysis

**Figure 1:**
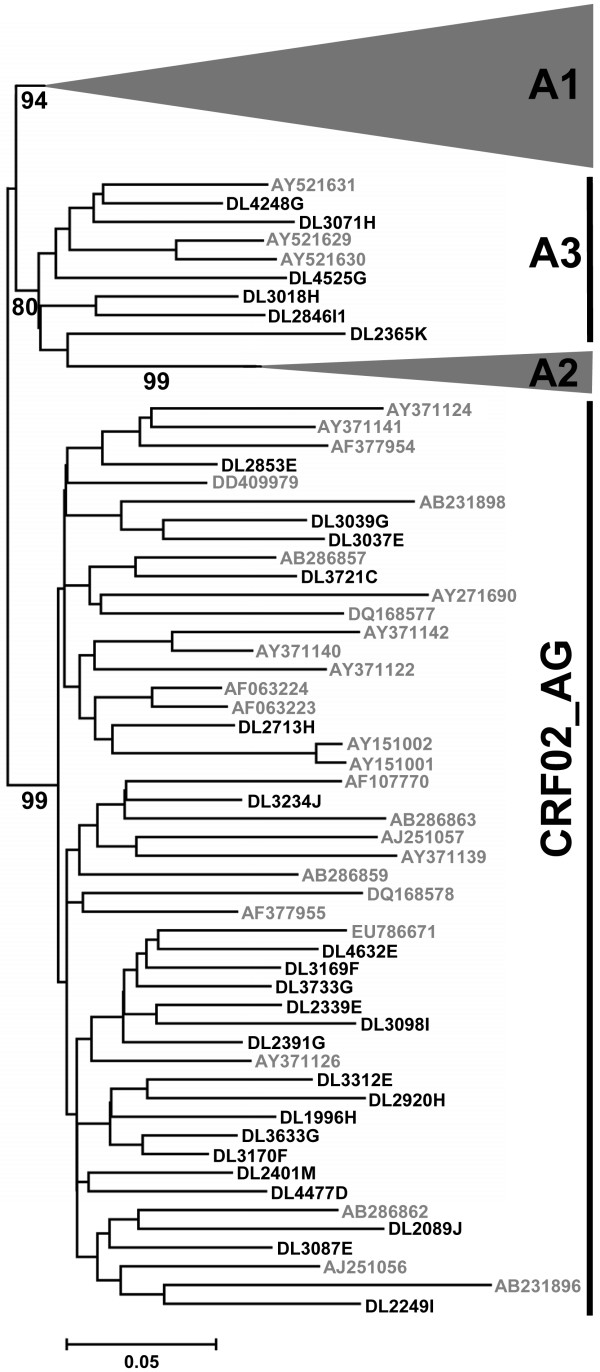
**Classification of subtype A-like sequences**. A neighbour joining tree showing detailed analysis of subtype A-like sequences. Interior branch test likelihoods (1000 bootstraps) are shown on branches distinguishing sub-subtypes and CRF02_AG. All sample sequences cluster within either the CRF02_AG or the A3 cluster. For visual clarity, subsubtype A1 and A2 clusters are represented by triangles.

Sequences were assembled, and contigs were analyzed with CodonCode Aligner version 1.5.2 (CodonCode Corporation, Dedham, USA) blinded to the phenotype. True permutated positions were detected by the software, and manually inspected. All sequences had open reading frames and were subjected for further analysis. A multiple alignment of our sequences with a reference sequence data set of all major subtypes, sub-subtypes and CRFs (downloaded from Los Alamos Sequence Database) was performed in MEGA4 using the Clustal algorithm [39-41]. Nucleotide sequences were aligned via protein sequences and major gap positions were removed to a final sequence length of 463 base pairs. A neighbor-joining (N-J) tree was constructed in MEGA4 using pair-wise deletion in a maximum composite likelihood substitution model with heterogeneous pattern among lineages with a gamma distribution of 0.8338 (Akaike Information Criterion (AIC), calculated by Modeltest [42]). The phylogenetic reconstruction was bootstrapped 1000 times to separate sequences of different subtypes. The gp120 V3 region of CRF02_AG is subtype A-derived, and separation of subclusters belonging to either CRF02_AG or subtype A were not possible in this tree. To further characterize these sequences, we constructed a reference data set consisting of sequences characterized as subtype A or CRF02_AG. To avoid any bias of previously incorrect subtyping due to similarities in the V3 region between subtype A and CRF02_AG, only HIV-1 full genome (> 8000 bp) sequences were allowed. We reconstructed an N-J tree with our Guinea-Bissau-derived sequences and the described reference sequences as outlined above. Reference sequences that formed separate monophyletic clusters were removed from the data set to obtain a final reconstruction distinguishing clusters of subsubtypes (A1-A3) and CRF02_AG (AIC, gamma distribution 0.9136) (Fig. 1). Since Felsenstein's bootstrap test can be too conservative, we used the bootstrap interior branch test, with 1000 bootstraps, which is a mathematically more rigorous statistical method for phylogenetic reconstructions of closely related sequences [43-45]. Details and accession numbers of the constructed reference sequence data set can be found in Additional file 1.

### Determination of coreceptor tropism and evaluation of phenotypic method

Human kidney embryonic 293T cells and human glioma U87.CD4 cells, stably expressing CD4 and one of the chemokine receptors (CCR5 or CXCR4) were maintained as previously described [46,47]. Chimeric viruses with patient-specific V1-V3 regions were generated based on the protocol from the Tropism Recombinant Test (TRT) with minor modifications [15,29]. Briefly, 500 ng of amplified V1-V3 fragments from each plasma sample and 3 μg of 43XCΔV, a NheI-linearized vector containing a full-length pNL4-3 genome with the V1-V3 region deleted, were transfected into 293T cells using the calcium phosphate precipitation method. Chimeric viruses were harvested and stored at -80°C. Twenty-four hours before infection, 10^5 ^U87.CD4 cells/well were seeded in 48 well plates. For infection, 500 μl of chimeric viruses were added in duplicate wells. Cells were washed three times with Dulbecco Modified Eagle Medium 16 hours post infection. Cultures were analyzed at day one, seven and nine for p24 antigen production by ELISA (Biomérieux, Boxtel, The Netherlands).

To evaluate the performance of the TRT on subtype A and CRF02_AG (which is A-like in the V1-V3 region of gp120), we used a panel of selected isolates (generously provided by Professor Jan Albert, Swedish Institute for Infectious Disease Control) (Table 1). Subtype and phenotype of the isolates had been determined previously [21]. To confirm the phenotype results, we used 200 μl of each isolate for infection of U87.CD4-CCR5 and U87.CD4-CXCR4 in duplicate wells. The cells were washed 16 hours post infection and then analyzed for p24 production as described. From each isolate the V1-V3 region was amplified and used to produce recombinant viruses for infection and analysis according to the described procedure. Infections of U87.CD4-CXCR4 with chimeric viruses derived from R5 isolates were used as negative controls to calculate the p24 background value of the assay. Infections with a significant increase (mean value +/- 3 S.D.) in p24 antigen production over time, compared to day one of the infection, were considered as positive infections.

### Genotypic characterization of CRF02_AG coreceptor use

The aim was to characterize genetic properties in HIV-1 V3 *env *distinguishing CRF02_AG with different coreceptor tropism. Only two sequences of the control panel (22480 and 22627) and three sequences of the plasma samples (DL4477D, DL2391G and DL22853E) were of HIV-1 CRF02_AG with pure CCR5 tropism. Since this number was too low for an appropriate comparison between the R5 and R5/X4 groups, we added all available V3 sequences, with known coreceptor tropism, from Los Alamos sequence data base, to a final dataset of 111 sequences (Additional files 2, 3, 4) [39]. Only one sequence per patient was subjected for analyses. Multiple alignments of CRF02_AG V3 amino acid sequences were performed as described, and positively [K and R] and negatively [D and E] charged amino acids were counted. In codons with amino acid mixtures, all possible permutations were assessed (Additional files 3 and 4). The combination resulting in the highest net charge was used for phenotype prediction. The performance of different sequence motif-based rules was measured in terms of sensitivity, specificity, positive predictive value (PPV) and negative predictive value (NPV). The sensitivity was determined as the fraction of predicted X4 sequences among the sequences from viruses phenotyped as CXCR4-using, the specificity as the fraction of predicted R5 sequences among the sequences from viruses phenotyped as CCR5-using only, the PPV as the fraction of correctly predicted X4 sequences among all predicted X4 sequences, and the NPV as the fraction of correctly predicted R5 sequences among all predicted R5 sequences.

### Statistics

All statistical analysis was performed using SPSS 16.0.

### Ethics

The study was approved by the Research Ethics Committee at the Karolinska Institute, Stockholm, and the Ministries of Health and the Interior in Guinea-Bissau.

### Nucleotide sequence accession numbers

Nucleotide sequences were deposited in GenBank under the following accession numbers: GQ401717-GQ401744, and FJ831886-FJ831893.

## Results

### Subtype determination

The HIV-1 V3 region from 29 plasma samples, collected during late-stage disease from 29 treatment-naïve individuals, was amplified and sequenced. A phylogenetic tree with these sequences and reference sequences of different subtypes was reconstructed. Sequences were well separated with long branches in the phylogeny, indicating patient-specific origin of sequences (Fig. 1). Twenty-eight sequences formed a subtype A cluster together with reference sequences of A1, A2 and CRF02_AG. The non-subtype A sequence clustered with reference strains of subtype C. The V3 region of CRF02_AG is subtype A-derived, and to further characterize these sequences we made a BLAST-search using each sample sequence as query sequence. The three most similar full genome (> 8000 bp) hits were used as reference sequences to distinguish subtype A from CRF02_AG. Among the 28 patients with subtype A-like sequences, 22 clustered with CRF02_AG reference sequences, and six with the previously described subsubtype A3 (Fig. 1, Table 2) [48].

### Accuracy of the phenotypic method for subtype A and CRF02_AG

Construction of chimeric viruses is a commonly used method for coreceptor tropism analysis, and both commercial and in-house variants can be found in the literature [29,30,49]. Here, we used for subtype B the well-established Tropism Recombinant Test (TRT) [29,50]. To confirm that the TRT assay performs equally well for HIV-1 subtype A and CRF02_AG as for HIV-1 subtype B, we used a control panel of HIV-1 subtype A and CRF02_AG isolates with known coreceptor tropism. We reanalyzed and confirmed previous results of the isolates by infecting the cell lines U87.CD4-CCR5 and U87.CD4-CXCR4, and by direct sequencing of the V3 region with flanking regions (Table 1). The V1-V3 region of each isolate was amplified and used for production of chimeric viruses. Tropism results of infections with chimeric viruses were in concordance with results from isolate infections, showing that the TRT assay can be used for determination of coreceptor tropism of HIV-1 subtype A and CRF02_AG (Table 1). The high concordance also suggests that the major determinants for coreceptor use are located within the V1-V3 region.

### Prevalence of HIV-1 CXCR4-using populations in subtype A and CRF02_AG infected individuals in late-stage disease

To investigate the prevalence of CXCR4-using populations in the 28 individuals from Guinea-Bissau infected with HIV-1 subtype A or CRF02_AG, we constructed infectious chimeric viruses with patient-specific gp120 V1-V3 regions. All chimeric viruses that were tested established productive infections with a significant increase in p24 antigen production over time (1-9 days) in U87.CD4-CCR5 and/or U87.CD4-CXCR4 cells. Twenty-one (75%) of the individuals studied had viruses that used both CCR5 and CXCR4 for cellular entry, whereas one (4%) and six (21%) individuals had pure X4 or R5 populations, respectively (Table 2). In subtype A infected individuals, three of six had CXCR4-using populations, whereas the corresponding number in CRF02_AG infected individuals were 19 of 22 (86%).

Recently, an evolving epidemic with increasing frequency of CXCR4 tropism among subtype C-infected individuals was suggested [11]. To determine if a similar pattern could be seen in our HIV-1 CRF02_AG material from Guinea-Bissau, we divided the data set in one early group (samples collected from 1997 to 2001), and one late group (samples collected from 2003 to 2007). In the early group, five out of eight samples had viruses that were CXCR4-tropic, whereas all samples in the late group were of this phenotype (14 out of 14) (p = 0.036, two-tailed Fisher's exact test). The two groups were well balanced with no significant differences in CD4+ T cell counts (p = 0.646 and p = 0.220 for CD4tot and CD4%, respectively, Mann-Whitney U test). To investigate if this difference could be found in a larger material of HIV-1 CRF02_AG we added all available data from the literature and in The Los Alamos Sequence Data Base where we could couple patient-specific disease status with coreceptor tropism and sampling year (Additional file 5). We found data of 43 different individuals sampled during 1997-2001 or 2003-2007 with defined late-stage disease and with known coreceptor tropism (42 from Cameroon and 1 from Ghana). When analyzed together with our samples from Guinea-Bissau, 28 out of 50 had viruses that were CXCR4-tropic in the early group, whereas 14 out of 15 were of this phenotype in the late group (p = 0.012, two-tailed Fisher's exact test) (Additional file 5).

### Molecular characterization of the V3 region coupled to coreceptor tropism

To create a data set that would allow for comparison between the R5 and R5X4/X4 groups, we combined the 22 CRF02_AG plasma-derived sequences with the three CRF02_AG sequences from the control panel and the 86 CRF02_AG sequences available in Los Alamos sequence data base with known phenotype resulting in a final dataset of 111 sequences (75 R5 and 36 R5X4 or X4 sequences) (Additional files 3 and 4). Sequences of subsubtype A3 were too few, and therefore not subjected for further analysis. Several sequence motif-based rules and bioinformatic tools have been developed to predict coreceptor tropism based on V3 sequences of HIV-1. These have mainly been based on subtype B, and the most common are the 11/25 rule (positively charged amino acids in V3 position 11 and/or 25 predicts CXCR4 tropism) and the net charge rule (a net charge of ≥+5 predicts CXCR4 tropism) [51]. Raymond *et al. *used the 11/25 rule in combination with the net charge rule to develop a genotypic rule specific for CRF02_AG (Table 3) [52]. Here, we used these rules and two widely used bioinformatic tools, WebPSSM and Geno2Pheno to predict the HIV-1 tropism based on the V3 sequence [32,53] (Table 3). The sensitivity reflects how many sequences of the true CXCR4-using viruses that the genotypic rule or bioinformatic tool identifies as CXCR4-using, whereas the specificity reflects how many sequences of the true CCR5-using viruses that the genotypic rule or bioinformatic tool identifies as CCR5-using. The PPV (R5X4/X4) and the NPV (R5) reflect how many of the predictions that are accurate. Of existing rules and tools, Geno2Pheno had the highest sensitivity, whereas the combined rule by Raymond *et al. *had the highest specificity (Table 3).

**Table 3:** Comparison of different genotypic rules and bioinformatic tools for prediction of HIV-1 coreceptor tropism based on the HIV-1 V3 amino acid sequence.

		No. patients with virus phenotype:		Performance (%)			
Prediction method	Predicted phenotype						
		R5	R5X4/X4	Sensitivity	Specificity	PPV^4^	NPV^5^
11/25	R5	71	25	31	95	73	74
	X4	4	11				
Net ≥ +5	R5	65	20	44	87	62	76
	X4	10	16				
Raymond *et al.*^1^	R5	73	22	39	97	88	77
	X4	2	14				
WebPSSM_X4R5_^2^	R5	63	22	39	84	54	74
	X4	12	14				
WebPSSM_SINSI_^2^	R5	69	25	31	92	65	73
	X4	6	11				
Geno2Pheno^3^	R5	66	18	50	88	67	79
	X4	9	18				
Net ≥ +4	R5	35	10	72	47	46	78
	X4	30	26				
Total ≥ 8	R5	63	18	50	84	60	78
	X4	12	18				
Net ≥ +5 and Total ≥ 8	R5	55	13	64	73	53	81
	X4	20	23				

^1^Raymond *et al. *used a combined rule were one of the following criteria was required for HIV-1 CRF02_AG CXCR4-tropism: (i) R or K at position 11 of V3 and/or K at position 25, (ii) R at position 25 of V3 and a net charge of ≥ + 5, or (iii) a net charge of ≥ + 6.

^2^Position-specific scoring matrix, http://indra.mullins.microbiol.washington.edu/webpssm/ (January 2010).

^3^Geno2Pheno was used with a false-positive rate of 10%. http://coreceptor.bioinf.mpi-inf.mpg.de/ (January 2010).

^4^Positive predictive value.

^5^Negative predictive value.

The sensitivity is of particular interest in clinical settings since it reflects the likelihood of detecting true CXCR4-tropism, and patients having HIV-1 of this tropism may not be suitable for treatment with coreceptor antagonists. Due to the low sensitivity of previous rules for HIV-1 of CRF02_AG, we used our data set to develop new rules. The mean net charges of V3 in the R5 and R5X4/X4 groups were 3.45 (95% CI: 3.27-3.64), and 4.47 (95% CI: 4.01-4.94), respectively. Modifying the net charge rule by setting the cutoff for prediction of CXCR4 tropism to ≥+4, we improved the sensitivity to 72%, although to a cost of both specificity (47%) and PPV (46%) (Table 3). We then examined another approach by counting the total number of charged amino acids. The mean numbers of charged amino acids were 6.60 (95% CI: 6.36-6.84) and 7.58 (95% CI: 7.16-8.01) for the R5 and R5X4/X4 group, respectively. Setting the cutoff to ≥ 8 charged amino acids for prediction of CXCR4 tropism resulted in a sensitivity equal to the one obtained by Geno2Pheno (50%) (Table 3).

Finally, we tested all possible combinations of previous genotypic rules analyzed in this study, and the net charge rule of ≥+4 and/or the total charge rule of ≥ 8 for prediction of CXCR4 use (data not shown). Of all combinations tested, the combination of the net charge rule of ≥+5 and total charge rule of ≥ 8 resulted in the best improvement in sensitivity (64%), without losing too much in specificity (73%) (Table 3).

## Discussion

In this study, we show that the V1-V3 region of the HIV-1 envelope is the major determinant for coreceptor tropism also for subtype A and CRF02_AG, and that the TRT assay accurately determines coreceptor tropism for these subtypes [29]. In our samples, we found a high prevalence of X4 populations (79%) during late-stage disease and an increasing frequency of HIV-1 with CXCR4 tropism in CRF02_AG-infected patients over time. We also demonstrate that the total number of charged amino acids may contribute to the development of genotypic rules and bioinformatic tools for prediction of coreceptor tropism of HIV-1 CRF02_AG.

HIV-1 subtype determination was done by amplifying and sequencing the envelope gp120 V3 region of 29 infected individuals in late-stage disease from Guinea-Bissau, West Africa. Twenty-eight of the individuals had HIV-1 of subtype A or CRF02_AG, confirming previous results that these are the dominating HIV-1 forms in Guinea-Bissau [54]. The remaining study subject was infected with HIV-1 subtype C, a subtype that has never been described in Guinea-Bissau before, even though it has been shown to circulate to some extent in West Africa [55]. A detailed analysis of the six patients infected with subtype A showed close genetic relationship to the previously described sub-subtype A3 (Fig. 1). This sub-subtype was first described in Senegal, but has been shown to be prevalent in several West African countries, including Guinea-Bissau [48,56].

A control panel of 11 HIV-1 subtype A and CRF02_AG isolates was used to show that the, for subtype B, well-characterized TRT assay is accurate also for subtype A and CRF02_AG. This finding suggests that the gp120 V1-V3 region is the major determinant of coreceptor phenotype also for subtype A and CRF02_AG. This conclusion is in line with the general concept previously established for subtype B that coreceptor phenotype is determined by the V1-V3 region of gp120, in particular the V3 region [23]. Our results show that the TRT can be used as a reliable alternative to the commercially available Trofile assay (Monogram Biosciences, San Francisco, USA), at least for the tested subtypes. The Trofile assay require amplification of the entire gp160 (2,500 bp), and it has been proposed that use of V1-V3 (940 bp) can be a more sensitive approach as it could reduce the risk of losing minority populations seen when amplifying a larger fragment [50].

Since we had a reliable phenotypic assay, we were able to analyze genetic traits that would best predict coreceptor phenotype for viruses from our HIV-1 CRF02_AG samples. Applying the 11/25 rule (positively charged amino acids in V3 position 11 and/or 25 predicts CXCR4 tropism) we found a sensitivity of 31%, and a specificity 95%. This is similar to a previous study of 113 CRF02_AG isolates, where the 11/25 rule had a sensitivity of 33%, and a specificity of 96% [13]. In another study the authors used the combined criteria of the 11/25 and the net charge rule of V3 sequences of 52 CRF02_AG isolates and found both high sensitivity (70%) and high specificity (98%) [52]. The results of the combined criteria could, however, not be verified by our data. A reason for the poor performance of this rule on our dataset may be that it relies on the 11/25 rule. In sequences from viruses with CXCR4 tropism from Guinea-Bissau we found in general negatively charged or non-charged amino acids in positions 11 and 25, whereas at least one of these positions in most cases were positively charged in the sequences used in the study by Raymond *et al*. (Additional files 3 and 4). We also observed many charged (both positive and negative) amino acids as a profound characteristic of sequences derived from CXCR4-using viruses (in the complete dataset). Therefore, we counted the total number of charged amino acids in the V3 region and combined it with the net charge rule. Due to sensitivity, this rule performed better than all of the analyzed rules and bioinformatic tools, without losing too much in specificity. This rule is also different to those rules that are used for subtype B, suggesting that molecular differences at the level of virus-cell receptor interaction may exist among HIV-1 subtypes. Further studies are needed to investigate subtype-specific genotypic differences involved in HIV-1 coreceptor tropism, and the sensitivity of existing genotypic rules and bioinformatic tools have to be increased before they can be used in a clinical setting.

Next, we examined the coreceptor phenotype of HIV-1 in 28 subtype A or CRF02_AG infected individuals in late-stage disease. CXCR4-using viral populations were found in as much as 79% of the analyzed samples, demonstrating the importance of analyzing samples from patients in late-stage disease when investigating HIV-1 subtype-specific predisposal for CXCR4 tropism. For HIV-1 subtype B, it is well known that CCR5 is the dominant coreceptor early in infection. Switch or broadening of coreceptor use from CCR5 to CXCR4 occurs late in disease [6]. To our knowledge, only one previous study has reported on CRF02_AG coreceptor tropism in late-stage disease [13]. Vergne *et al. *found that 56% of the studied isolates were positive for MT-2 cell tropism, to be compared to our result of CXCR4 use of 86%. Although, it is important to note that the viruses in the study by Vergne *et al. *were isolated from samples collected between 1996 and 2001, and the corresponding number of CXCR4-tropism among our samples during this time-frame was 63%.

Connell *et al. *(2008) analyzed the results of 19 subtype C isolates, isolated in 2005, and found a higher prevalence (30%) of CXCR4 tropism than has been shown in earlier studies [11]. They suggested that HIV-1 subtype C might be an evolving epidemic, showing an increasing prevalence of CXCR4 phenotype over time in South Africa. In the present study, we performed a direct comparison by dividing our 22 CRF02_AG samples into two groups: Samples from 1997 to 2001, and samples from 2003 to 2007. The CD4+ T cell counts and clinical data were similar between the two groups, excluding any bias due to differences in disease status. In addition, all of the investigated individuals were treatment naïve. We found a significant difference between the groups, suggesting a similar kind of evolving epidemic for CRF02_AG in Guinea-Bissau that has been suggested for subtype C in South Africa. We also analyzed our data together with available data of HIV-1 CRF02_AG infected patients with known clinical parameters and coreceptor tropism and found that the trend of an evolving epidemic was consistent. Competition assays between HIV-1 R5 and X4 viruses have shown that X4 viruses in general out-compete R5 viruses due to both higher replication kinetics and higher CXCR4 than CCR5 expression in PBMC [57]. Moreover, it has been shown that sequence change occur at a rate of 1% per year in HIV-1 *env*, illustrating the constant evolution of HIV-1 on the genetic level [58]. Further studies (based on larger sample sizes than studied here and by Connell *et al.*) are needed to investigate if HIV-1 is evolving towards a more predisposed state of changing into CXCR4 phenotype on a population level, and a confirmation of this finding in larger cohorts may have implications for viral transmission, pathogenesis and disease progression.

**Figure 2:**
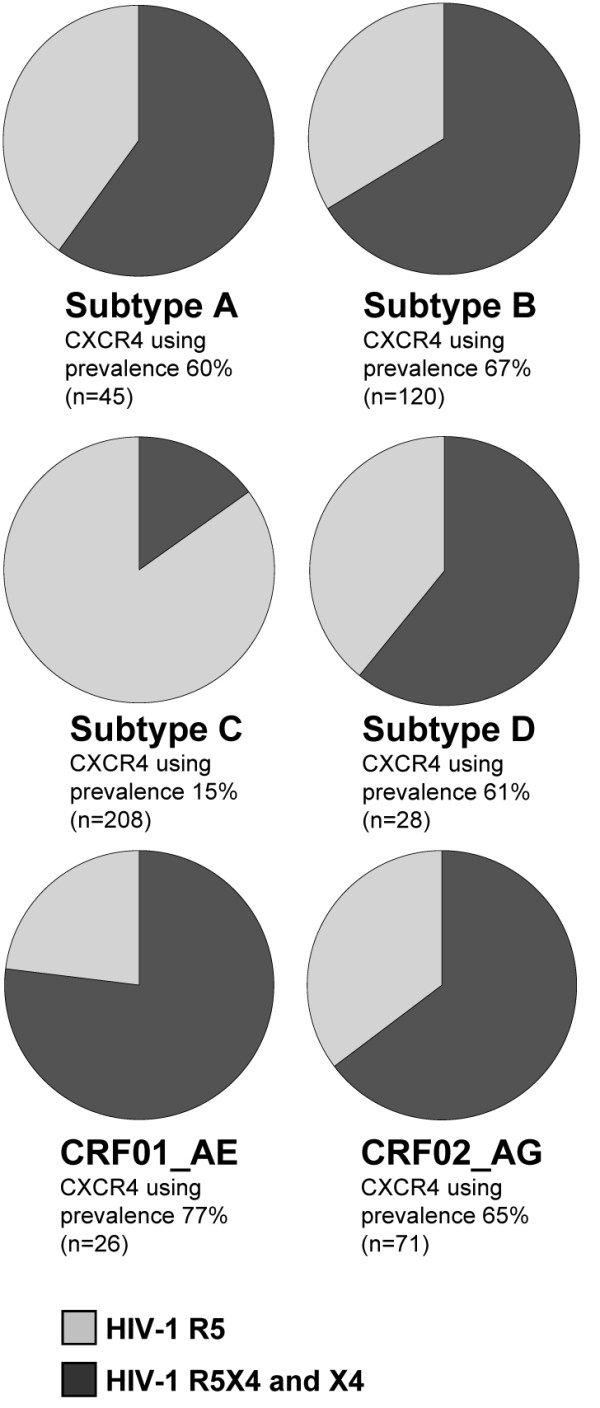
**Prevalence of HIV-1 CXCR4-tropism in late-stage disease in different subtypes**. Combined results of new data presented in this study and data from the literature analysis, showing the prevalence of HIV-1 CXCR4-tropic viruses in late stage disease. The number of individuals used in each diagram is specified within brackets. A detailed description of the analysis can be found in Additional file 6.

To further the view of our results, we performed a literature review of published results regarding subtype-specific coreceptor tropism in late-stage disease, which, to our knowledge, has not been presented before. The analysis included 498 patient-specific HIV-1 samples of six different subtypes, sampled over more than 20 years (1988-2008). Data of CD4+ T cell counts and/or clinical status for all patients were examined, and only samples from individuals diagnosed with AIDS or having CD4+ T cell counts ≤ 200 cells/μl were included in the analysis. In cases were both CD4+ T cell counts and clinical status could be found, the criterion of CD4+ T cell count was used. The vast majority of patients were included based on CD4+ T cell counts. Moreover, only one sample per patient was allowed, and in cases where the same patients appeared in several studies the patient data were only used once (for details about the analysis, see Additional file 6). HIV-1 coreceptor tropism determined as MT-2 non-syncytium or syncytium inducing was regarded as CCR5 or CXCR4 tropism, respectively [22]. If HIV-1 subtype and coreceptor tropism were not specified, this data were confirmed by personal communication with the authors (Additional file 6, and Acknowledgement). Our analysis revealed a high frequency of HIV-1 R5X4 or X4 populations in late stage disease among all analyzed subtypes, except for subtype C (Fig. 2). Only 15% of the individuals infected with HIV-1 subtype C had CXCR4-using populations, compared to 66% (60%-77%) in individuals infected with HIV-1 of non-subtype C (p < 0.001, two-tailed Fisher's exact test).

The data from the literature review also allowed us to investigate if we could confirm the results of Connell *et al. *of an evolving epidemic in South Africa, and if this could be seen for subtype C in general [11]. We divided the subtype C data set (208 patients, samples collected in Cameroon, Ethiopia, India, Malawi, South Africa, Sweden and Zimbabwe) into an early group (samples before the year of 2000) and a late group (samples after 2000) (Additional file 7). In the early group from South Africa eight of 46 patients (17%) had CXCR4-using viruses, whereas the corresponding number in the late group was 11 of 36 (31%) (p = 0.19, two-tailed Fisher's exact test). Connell *et al. *reported that 30% of their isolates were able to use CXCR4 and compared this to previous studies from South Africa showing no syncytium-inducing (SI) capacity of HIV-1 isolates collected during the 1980s, whereas 10-17% of the studied isolates had SI capacity during the 1990s. No statistical evaluation was performed in their study. In the early group of our complete subtype C data set, 11 of 145 patients (8%) had CXCR4-using viruses, whereas the corresponding number in the late group was 21 of 63 (33%) (p < 0.001, two-tailed Fisher's exact test). Our results confirms the numbers presented by Connell *et al*. in South Africa (even though the difference between the early and the late group was not statistically significant), and further strengthens the concern of an evolving HIV-1 subtype C-epidemic on the population level.

Understanding subtype-specific differences regarding coreceptor tropism is important for several reasons. First, these studies may help us understanding differences in HIV-1 pathogenesis. Several studies have indicated differences in relative pathogenicity between different subtypes, where subtype D appears to be more pathogenic compared to other subtypes [59]. Moreover, it has been shown that HIV-1 subtype D has a preference for CXCR4 tropism early in infection, and a connection with the faster disease progression seen in this subtype has been suggested [3]. Second, understanding HIV-1 subtype-specific differences regarding the ability to develop CXCR4-using populations may be of great importance for future treatment guidelines for coreceptor antagonists. Fätkenheuer *et al. *(2008) reported a striking difference in the appearance of X4 populations among patients with experienced treatment failure, after receiving either the CCR5-antagonist Maraviroc or a placebo treatment [60]. Despite a short follow-up period of only 48-weeks, as many as 57% of patients receiving Maraviroc developed X4 viruses (76 of 133 patients), compared to only 6% (6 of 95 patients) in the placebo group. This finding strengthens the concerns that CCR5 antagonists could increase the risk of HIV-1 populations to shift away from CCR5 to CXCR4 use, potentially leading to treatment failure and faster disease progression [61,62]. Most of the participants in the above study were infected with HIV-1 subtype B, and more studies are needed to determine if the outgrowth of R5X4 or X4 populations will be as distinct also for other subtypes, such as subtype C.

Based on the accumulated knowledge on subtype C infections, it is tempting to speculate that subtype C-infected patients in general may be more suitable for treatment with CCR5 antagonists than patients infected with other subtypes, at least in late-stage disease. Taken these thoughts further, different subtypes and CRFs may be more or less predisposed for the emergence of R5X4 or X4 populations, although it seems that CXCR4-using HIV-1 populations likely will arise in most HIV-1 non-subtype C infections (Fig. 2). The comparative picture of subtype-dependent HIV-1 coreceptor tropism in late-stage disease underlines the importance of further studies on HIV-1 subtype-dependent coreceptor tropism, an issue of direct clinical importance for the development of treatment guidelines of the recently introduced coreceptor antagonists in HIV treatment regimens.

## Conclusion

In summary, we show that the TRT assay accurately determines the HIV-1 coreceptor tropism of subtype A and CRF02_AG. Using this assay we found a high amount of HIV-1 CXCR4-using populations (79%) in our plasma samples and an increasing frequency of CXCR4 tropism in CRF02_AG-infected patients over time. The emergence of CXCR4 use may have implications for viral transmission, pathogenesis and disease progression. We also demonstrate that the combined criteria of the total number of charged amino acids and net charge of the V3 region is a more sensitive predictor of CXCR4 tropism for HIV-1 CRF02_AG compared to the analyzed genotypic rules and bioinformatic tools. Finally, we conducted an extensive literature analysis of 498 individuals which, to our knowledge, is the most extensive comparison of subtype-specific coreceptor tropism in late-stage disease. We found a generally high frequency of CXCR4 tropism among all major subtypes and CRFs, except for subtype C. These results demonstrate the need for further studies investigating subtype-specific emergence for CXCR4-tropism, this may be particularly important due to the introduction of CCR5-antagonists in HIV treatment regimens.

## Competing interests

The authors declare that they have no competing interests.

## Authors' contributions

JE designed the study, optimized the experimental protocols, performed RNA extraction and sequencing, analyzed and interpreted the data, performed the literature analysis, and wrote the manuscript. FM contributed in study design, clinically evaluated the patient data and participated in patient selection. WMA performed most of the cloning and colony-PCR, and participated in optimization of the cloning strategy. EV carried out cell assays, ELISA experiments, and participated in optimization of the TRT assay. AJB was medically and organizationally responsible for the clinical sites with biological samples of the study participants in the cohort. ZJdS was responsible for analyses of HIV serology at the laboratory in Guinea-Bissau. EMF participated in interpretation of the results, and contributed to the literature analysis. HN contributed in study design, and participated in patient selection. PM designed the study, participated in analyzing and in interpretation of the data, and helped to draft the manuscript. All authors read and approved the manuscript.

## Supplementary Material

Additional file 1 **Table S1 - Reference dataset of subsubtype A1-A3 and CRF02_AG sequences**. HIV-1 reference dataset of subsubtype A1-A3 and CRF02_AG sequences used in the final phylogenetic reconstruction for subsubtype and CRF02_AG determination of the sample sequences. Click here for file

Additional file 2 **Table S2 - Reference dataset of CRF02_AG sequences used in the molecular analysis**. Accession numbers of the HIV-1 reference dataset of HIV-1 CRF02_AG sequences with phenotypically determined coreceptor tropism used in the molecular analysis. Click here for file

Additional file 3 **Table S3 - Alignment and molecular characteristics of HIV-1 CRF02_AG V3 amino acid sequences from study samples and references with determined CCR5 tropism**. Summary of the molecular characteristics of the CCR5 tropic sequences used in the genotypic analysis. Click here for file

Additional file 4 **Table S4 - Alignment and molecular characteristics of HIV-1 CRF02_AG V3 amino acid sequences from study samples and references with determined CXCR4 tropism**. Summary of the molecular characteristics of the CXCR4 tropic sequences used in the genotypic analysis. Click here for file

Additional file 5 **Table S5. Data used to investigate an evolving epidemic available data from HIV-1 CRF02_AG**. Summary of the data obtained from the literature and Los Alamos Sequence Data Base to investigate if the HIV-1 CRF02_AG epidemic represents an evolving epidemic. Click here for file

Additional file 6 **Table S6 - Overview of the literature analysis**. Summary of the data obtained from the literature and used to determine the amount of CXCR4 tropism in late stage disease in the studied HIV-1 subtypes. Click here for file

Additional file 7 **Table S7 - Overview of the subtype C material**. Summary of the subtype C data obtained from the literature review used in the analysis of an evolving epidemic for subtype C. Click here for file
