# Supplementary material for: Frequent CXCR4 tropism of HIV-1 subtype A and CRF02_AG during late-stage disease - indication of an evolving epidemic in West Africa
Source: Retrovirology. 2010 Mar 22;7:23. doi: 10.1186/1742-4690-7-23 (PMC2855529; doi:10.1186/1742-4690-7-23)
Supplement: Additional file 1 — Table S1 - Reference dataset of subsubtype A1-A3 and CRF02_AG sequences. HIV-1 reference dataset of subsubtype A1-A3 and CRF02_AG sequences used in the final phylogenetic reconstruction for subsubtype and CRF02_AG determination of the sample sequences. [file 1742-4690-7-23-S1.DOC]

**Additional Table S1. HIV-1 reference dataset of subsubtype A1-A3 and CRF02_AG sequences used in the final phylogenetic reconstruction for subsubtype and CRF02_AG determination of the sample** sequences.

| AB098332 | AF063223 | AF361873 | AF457079 | AF539405 | AY371124 | DQ083238 |
| --- | --- | --- | --- | --- | --- | --- |
| AB231896 | AF063224 | AF377954 | AF457081 | AJ251056 | AY371126 | DQ168577 |
| AB231898 | AF069669 | AF377955 | AF457083 | AJ251057 | AY371139 | DQ168578 |
| AB253421 | AF069670 | AF457052 | AF457084 | AY151001 | AY371140 | EU110087 |
| AB253428 | AF069673 | AF457055 | AF457086 | AY151002 | AY371141 | EU110088 |
| AB286857 | AF107770 | AF457063 | AF457089 | AY253314 | AY371142 | EU110092 |
| AB286859 | AF107771 | AF457065 | AF484478 | AY271690 | AY521629 | EU110094 |
| AB286862 | AF286237 | AF457066 | AF484493 | AY322184 | AY521630 | EU786671 |
| AB286863 | AF286238 | AF457067 | AF484507 | AY322190 | AY521631 |  |
| AB287376 | AF286241 | AF457069 | AF484508 | AY322193 | AY713406 |  |
| AF004885 | AF361872 | AF457075 | AF484509 | AY371122 | DD409979 |  |
