# Supplementary material for: Frequent CXCR4 tropism of HIV-1 subtype A and CRF02_AG during late-stage disease - indication of an evolving epidemic in West Africa
Source: Retrovirology. 2010 Mar 22;7:23. doi: 10.1186/1742-4690-7-23 (PMC2855529; doi:10.1186/1742-4690-7-23)
Supplement: Additional file 2 — Table S2 - Reference dataset of CRF02_AG sequences used in the molecular analysis. Accession numbers of the HIV-1 reference dataset of HIV-1 CRF02_AG sequences with phenotypically determined coreceptor tropism used in the molecular analysis. [file 1742-4690-7-23-S2.DOC]

**Additional Table S2. Accession numbers of the HIV-1 reference dataset of HIV-1 CRF02_AG sequences with phenotypically determined coreceptor tropism used in the molecular analysis.**

| AB049811 | AM279352 | AY371127 | FJ652327 | FJ652340 | FJ652354 | FJ652370 |
| --- | --- | --- | --- | --- | --- | --- |
| AF063223 | AM279356 | AY371128 | FJ652328 | FJ652341 | FJ652355 | FJ652371 |
| AF119216 | AM279358 | AY371138 | FJ652329 | FJ652342 | FJ652356 | FJ652373 |
| AF184155 | AM279360 | AY736839 | FJ652330 | FJ652343 | FJ652357 | FJ652374 |
| AF355318 | AM279361 | AY736840 | FJ652331 | FJ652344 | FJ652359 | FJ652375 |
| AF355320 | AM279362 | AY994510 | FJ652332 | FJ652345 | FJ652362 | FJ652377 |
| AF355321 | AM279367 | DQ177193 | FJ652333 | FJ652346 | FJ652363 | FJ652378 |
| AF355325 | AY271690 | DQ177209 | FJ652334 | FJ652347 | FJ652364 | L22939 |
| AF355327 | AY371122 | DQ825459 | FJ652335 | FJ652348 | FJ652365 |  |
| AF355331 | AY371123 | DQ825460 | FJ652336 | FJ652349 | FJ652366 |  |
| AF355334 | AY371124 | DQ825462 | FJ652337 | FJ652350 | FJ652367 |  |
| AF355335 | AY371125 | DQ825471 | FJ652338 | FJ652351 | FJ652368 |  |
| AF355336 | AY371126 | DQ869018 | FJ652339 | FJ652353 | FJ652369 |  |
