# Supplementary material for: Frequent CXCR4 tropism of HIV-1 subtype A and CRF02_AG during late-stage disease - indication of an evolving epidemic in West Africa
Source: Retrovirology. 2010 Mar 22;7:23. doi: 10.1186/1742-4690-7-23 (PMC2855529; doi:10.1186/1742-4690-7-23)
Supplement: Additional file 3 — Table S3 - Alignment and molecular characteristics of HIV-1 CRF02_AG V3 amino acid sequences from study samples and references with determined CCR5 tropism. Summary of the molecular characteristics of the CCR5 tropic sequences used in the genotypic analysis. [file 1742-4690-7-23-S3.DOC]

**Additional Table S3 – Alignment and molecular characteristics of HIV-1 CRF02_AG V3 amino acid sequences from study samples and references with determined CCR5 tropism.**

| **R5** | | | | | | | | | |
| --- | --- | --- | --- | --- | --- | --- | --- | --- | --- |
| **Set^1^** | **Sample^2^** | **V3 sequence^3^** | **Length** | **Position^4^** | | **Charge^5^** | | | |
|  |  | \| \| \|\| \| \| \|\| \| |  | 11 | 25 | + | - | Net | Total |
| GB | DL2391G | CTRPNNNTRKSIGIGPGQTFYAAGEIIGDIRRAHC | 35 | 0 | - | 5 | 2 | 3 | 7 |
|  |  | RGV |  |  |  |  |  |  |  |
| GB | DL2853E | CIRPNNNTRKSIRIGPGQTFYARGDIIGDIRQAHC | 35 | 0 | - | 5-6 | 2 | 3-4 | 7-8 |
|  |  | V T Y |  |  |  |  |  |  |  |
| GB | DL4477D | CTRPGNNTRKGVHMGTGKTFYATGDIIGDIRQAHC | 35 | 0 | - | 5 | 2 | 3 | 7 |
| Control | 22480 | CIRPNNNTRKSIRIGPGQTFYAT-DIIGNIRQAHC | 35 | 0 | - | 5 | 1 | 4 | 6 |
| Control | 22627 | CTRPGNNTRQSVRIGPGQTFYARGDITGDIRQAHC | 35 | 0 | - | 5 | 2 | 3 | 7 |
| LASDB | AB049811 | CTRPNNNTRKSVRIGPGQTFYATGDIIGDIRQAHC | 35 | 0 | - | 5 | 2 | 3 | 7 |
| LASDB | AF063223 | CPRPNNNTRKSVRIGPGQTFYATGDIIGDIRQAHC | 35 | 0 | - | 5 | 2 | 3 | 7 |
| LASDB | AF119216 | CTKPNNNTRKSVRIGPGQTFYASGDIVGNIRQAHC | 35 | 0 | - | 5 | 1 | 4 | 6 |
| LASDB | AF184155 | CTRPNNNTRKSVRIGPGQTFYATGGIIGDIRQAHC | 35 | 0 | 0 | 5 | 1 | 4 | 6 |
| LASDB | AF355318 | CTRPNNNTRKSVRIGPGQTFYATGEVIGDIRQAHC | 35 | 0 | - | 5 | 2 | 3 | 7 |
| LASDB | AF355321 | CTRPGNNTRKSVRIGPGQTFYATGDIIGDIRQAHC | 35 | 0 | - | 5 | 2 | 3 | 7 |
| LASDB | AF355325 | CTRPNNNTRKRVPIGPGQTFYAT-DIIGNIRQAHC | 34 | + | - | 5 | 1 | 4 | 6 |
| LASDB | AF355327 | CIRPNNNAIKNVGIGPGQTFYTTGKIIGNISQAYC | 35 | 0 | + | 3 | 0 | 3 | 3 |
| LASDB | AF355331 | CTRPNNNTRTSVRIGPGQTFYATGAIIGDIRKAYC | 35 | 0 | 0 | 5 | 1 | 4 | 6 |
| LASDB | AF355335 | CTRPHNNTRTSTRIGPGQTFYATGDIIGDIRQAHC | 35 | 0 | - | 4 | 2 | 2 | 6 |
| LASDB | AM279352 | CTRVANNTRTSVRIGPGQTFYATGGIIGDIRQAHC | 35 | 0 | 0 | 4 | 1 | 3 | 5 |
| LASDB | AM279358 | CTRPDNNTRKSVRIGPGQAFY-TNEIIGDIRKAHC | 34 | 0 | - | 6 | 3 | 3 | 9 |
| LASDB | AM279360 | CSRPGNNTRQSVRIGPGQTFYATGEIIGDIRQAHC | 35 | 0 | - | 4 | 2 | 2 | 6 |
| LASDB | AM279361 | CTRPNNNTRRSVRIGPGQTFYAAGEIIGDIRQAHC | 35 | 0 | - | 5 | 2 | 3 | 7 |
| LASDB | AM279362 | CTRPNNNTRKSVRIGPGQAFYTTTDITGDIRQAHC | 35 | 0 | - | 5 | 2 | 3 | 7 |
| LASDB | AM279367 | CTRPGNNTRKSVRIGPGQTFYATGDIIGDIRQAHC | 35 | 0 | - | 5 | 2 | 3 | 7 |
| LASDB | AY371122 | CTRPSNNTRKSIRIGPGQTFYATNNIIGNIRQAHC | 35 | 0 | 0 | 5 | 0 | 5 | 5 |
| LASDB | AY371123 | CTRPGNNTRKSVRIGPGQTFYATGDIIGDIRKAHC | 35 | 0 | - | 6 | 2 | 4 | 8 |
| LASDB | AY371124 | CIRPSNNTRKSIRIGPGQTFYATGAIIGDIRRAHC | 35 | 0 | 0 | 6 | 1 | 5 | 7 |
| LASDB | AY371125 | CTRPSNNTRTSVRIGPGQTFYGTGEIIGDIRKAYC | 35 | 0 | - | 5 | 2 | 3 | 7 |
| LASDB | AY371126 | CTRPGNNTRKSVRIGPGQTFYASGAIIGDIRQSHC | 35 | 0 | 0 | 5 | 1 | 4 | 6 |
| LASDB | AY371127 | CTRPNNNTRKSVRIGPGQTFYATGEIVGNIRQAYC | 35 | 0 | - | 5 | 1 | 4 | 6 |
| LASDB | AY371128 | CVRPGNNTIRSIRIGPGQSFHGTGNIIGDIRQAHC | 35 | 0 | 0 | 4 | 1 | 3 | 5 |
| LASDB | AY371138 | CTRTGKNTRTSIHMGPGQSAFFAGEVIRDIRLAYC | 35 | 0 | - | 5 | 2 | 3 | 7 |
| LASDB | AY736839 | CTRPNNNTRKSVRIGPGQTFYATGDIIGDIRQAHC | 35 | 0 | - | 5 | 2 | 3 | 7 |
| LASDB | AY736840 | CTRPNNNTRKSVRIGPGQTFYATGDIIGNIRQAYC | 35 | 0 | - | 5 | 1 | 4 | 6 |
| LASDB | AY994510 | CTRPSNNTRKGWHIGPGQTLYATGAIIGDIRQAHC | 35 | 0 | 0 | 4 | 1 | 3 | 5 |
| LASDB | DQ177193 | CTRPNNNTRRDIGIGPGQTFFAAGAIIGDIRQASC | 35 | - | 0 | 4 | 2 | 2 | 6 |
| LASDB | DQ177209 | CIRPNNNTRRSVRIGPGQTFYATGDIIGDIRQAYC | 35 | 0 | - | 5 | 2 | 3 | 7 |
| LASDB | DQ825471 | CTRPGNNTRQSIRIGPGQTFYATGDIIGDIRKAFC | 35 | 0 | - | 5 | 2 | 3 | 7 |
|  |  | V |  |  |  |  |  |  |  |
| LASDB | DQ869018 | CTRPGNNTRKSVRIGPGQTFYATGDITGDIRQAHC | 35 | 0 | - | 5 | 2 | 3 | 7 |
| LASDB | FJ652327 | CTRPNNNTRKSVRIGPGQTFYATGGIIGDIRQAHC | 35 | 0 | 0 | 5 | 1 | 4 | 6 |
|  |  | Y |  |  |  |  |  |  |  |
| LASDB | FJ652328 | CTRPGNNTRKSVRIGPGQTFYATGDIIGDIRQAHC | 35 | 0 | - | 5 | 2 | 3 | 7 |
| LASDB | FJ652329 | CIRPGNNTSKGIHIGPGRTFFANDRIIGDIRSAHC | 35 | 0 | + | 5 | 2 | 3 | 7 |
| LASDB | FJ652330 | CIRPGNNTRKSMRIGPGQTFYATGDIIGDIRQAHC | 35 | 0 | - | 5 | 2 | 3 | 7 |
| LASDB | FJ652331 | CTRPNNNTRKSIHIGPGRAFYATGDVIGNPRQAHC | 35 | 0 | - | 5 | 1-3 | 2-4 | 6-8 |
|  |  | M D T D K |  |  |  |  |  |  |  |
| LASDB | FJ652332 | CIRPNNNIRKSVRIGPGQTFYATGEIIGDIRKAHC | 35 | 0 | - | 4-6 | 2 | 2-4 | 6-8 |
|  |  | S T T Q Y |  |  |  |  |  |  |  |
| LASDB | FJ652333 | CMRPNNNTRKSVRIGPGQTFYAT-DIIGKIRQAYC | 34 | 0 | - | 5-6 | 1-2 | 3-5 | 6-8 |
|  |  | N E |  |  |  |  |  |  |  |
| LASDB | FJ652335 | CTRPSNNTRKSVHIGPGQTFYATGQIIGDIRQAHC | 35 | 0 | 0 | 4 | 1 | 3 | 5 |
|  |  | G P T |  |  |  |  |  |  |  |
| LASDB | FJ652336 | CIRPGNNTRKSGRRGPGQAFYATGDIIGDIRQAHC | 35 | 0 | - | 5-6 | 2-3 | 2-4 | 7-9 |
|  |  | V I D T |  |  |  |  |  |  |  |
| LASDB | FJ652337 | CTRPANKTIKGVRIGPGQTFYTTGSIIGNIRQAHC | 35 | 0 | 0 | 4-5 | 0-1 | 3-5 | 4-6 |
|  |  | N D Y |  |  |  |  |  |  |  |
| LASDB | FJ652338 | CTRPHNNTRKSVHIGPGQAFYATGDIIGDIRQAHC | 35 | 0 | - | 4 | 2 | 2 | 6 |
| LASDB | FJ652339 | CTRPNNNTRRSVRIGPGQAFYATGDIIGNIRQAHC | 35 | 0 | - | 5 | 1-2 | 3-4 | 6-7 |
|  |  | K A T D K Y |  |  |  |  |  |  |  |
|  |  | A |  |  |  |  |  |  |  |
|  |  | V |  |  |  |  |  |  |  |
| LASDB | FJ652340 | CTRPGNNTRQSVRIGPGQVFYA-NPIIGDIRQAHC | 34 | 0 | 0 | 4 | 1 | 3 | 5 |
| LASDB | FJ652341 | CVRPNNNTRKSVRIGPGQTFYATGDIRGDIRQAYC | 35 | 0 | - | 5-6 | 2 | 3-4 | 7-8 |
|  |  | T |  |  |  |  |  |  |  |
|  |  | S |  |  |  |  |  |  |  |
|  |  | I |  |  |  |  |  |  |  |
|  |  | L |  |  |  |  |  |  |  |
| LASDB | FJ652342 | CTRPNNNTRKSVRIGPGQAFYAT-DIIGDIRQAYC | 34 | 0 | - | 5 | 2 | 3 | 7 |
| LASDB | FJ652343 | CTRPGNNTRKSVRIGPGQTFYATGDIIGDIRQAHC | 35 | 0 | - | 5 | 2 | 3 | 7 |
| LASDB | FJ652344 | CIRPNNNTRKSVRIGPGQTFYAAGEIIGNIRQAHC | 35 | 0 | - | 5 | 1 | 4 | 6 |
|  |  | G |  |  |  |  |  |  |  |
| LASDB | FJ652345 | CTRPNNNTRKSIRIGPGQTFYATGDIIGDIRQAHC | 35 | 0 | - | 5 | 2 | 3 | 7 |
| LASDB | FJ652346 | CIRPNNNTRKSVHIGPGQAFYATNDIIGDIRQAHC | 35 | 0 | - | 4 | 2 | 2 | 6 |
|  |  | G PM |  |  |  |  |  |  |  |
| LASDB | FJ652347 | CTRPGNNTRTSVRIGPGQAFYATGDIIGDIRKAHC | 35 | 0 | - | 5 | 2 | 3 | 7 |
|  |  | L |  |  |  |  |  |  |  |
| LASDB | FJ652348 | CTRLNNNTRTSIRIGPGQSFHATGQIVGDIRQAHC | 35 | 0 | 0 | 4 | 1 | 3 | 5 |
| LASDB | FJ652349 | CIRPNNNTRKSVRIGPGQTFYATGDIIGNIRKAYC | 35 | 0 | - | 6 | 1-2 | 4-5 | 7-8 |
|  |  | D K |  |  |  |  |  |  |  |
| LASDB | FJ652350 | CIRPNNNKRRSVRIGPGQTFYATGDIIGNIRQAYC | 35 | 0 | - | 6 | 1 | 5 | 7 |
| LASDB | FJ652351 | CTRPGNNTRKSMRIGPGQTFYAYGEIIGDIRQAYC | 35 | 0 | - | 4-5 | 2 | 2-3 | 6-7 |
|  |  | G H V |  |  |  |  |  |  |  |
| LASDB | FJ652353 | CIRPGNNKRTSIRIGPGQTFYATGEIIGNIRQAQC | 35 | 0 | - | 5 | 1 | 4 | 6 |
|  |  | S |  |  |  |  |  |  |  |
| LASDB | FJ652354 | CVRPNNNTRKSVRIGPGQTFYATGEIIGDIRQAHC | 35 | 0 | - | 4-5 | 2-3 | 1-3 | 6-8 |
|  |  | G H DD K |  |  |  |  |  |  |  |
| LASDB | FJ652356 | CSRPNNNTRKSVRIGPGQTFYAT-DIIGDIRQAHC | 34 | 0 | - | 4-5 | 2-3 | 1-3 | 6-8 |
|  |  | V I PP |  |  |  |  |  |  |  |
|  |  | Q |  |  |  |  |  |  |  |
|  |  | E |  |  |  |  |  |  |  |
| LASDB | FJ652357 | CTRPNNNTRKSVRIGPGQTFYATGDIIGNIRQAHC | 35 | 0 | - | 5 | 1-3 | 2-4 | 6-8 |
|  |  | A G E D K |  |  |  |  |  |  |  |
|  |  | S |  |  |  |  |  |  |  |
|  |  | E |  |  |  |  |  |  |  |
| LASDB | FJ652359 | CTRPGNNTRKSVRIGPGQTFFATGAIIGNIRQAHC | 35 | 0 | -/0 | 5 | 0-2 | 3-5 | 5-7 |
|  |  | A D T D |  |  |  |  |  |  |  |
|  |  | I |  |  |  |  |  |  |  |
|  |  | V |  |  |  |  |  |  |  |
| LASDB | FJ652362 | CTRPNNNTRRSVRIGPGQTFYATGGIIGNIRQAHC | 35 | 0 | -/0 | 5 | 0-2 | 3-5 | 5-7 |
|  |  | I K A D |  |  |  |  |  |  |  |
|  |  | D |  |  |  |  |  |  |  |
| LASDB | FJ652363 | CTRPSNNTIKGIHIGPGRAFYTTGQIIGDIRQAHC | 35 | 0 | 0 | 4 | 1 | 3 | 5 |
| LASDB | FJ652364 | CTRPSNNTIKGIHIGPGRAFYTTGQIIGDIRQAHC | 35 | 0 | 0 | 4 | 1 | 3 | 5 |
|  |  | V F |  |  |  |  |  |  |  |
| LASDB | FJ652365 | CTRPNNNTRKSVHLGPGQAFYATGDIIGDIRQAYC | 35 | 0 | - | 4 | 2 | 2 | 6 |
| LASDB | FJ652366 | CIRPGNNTRKSVRIGPGQAFYATGAVAGDIRQAHC | 35 | 0 | 0 | 5 | 1 | 4 | 6 |
| LASDB | FJ652367 | CIRPGNNTRKSVRIGPGQTFYATGDIIGDIRQAHC | 35 | 0 | - | 5 | 2 | 3 | 7 |
| LASDB | FJ652368 | CTRPNNNTRTSIRIGPGQTFYANNGIIGDIRQAHC | 35 | 0 | 0 | 4 | 1 | 3 | 5 |
| LASDB | FJ652371 | CTRPNNNTRKSVRIGPGQTFYATGEIIGKIRQAHC | 35 | 0 | - | 4-6 | 1 | 3-5 | 5-7 |
|  |  | I T D N |  |  |  |  |  |  |  |
| LASDB | FJ652378 | CTRPGNNTSKSVRIGPGQVFYATGRIIGNIRQAHC | 35 | 0 | + | 5 | 0-1 | 4-5 | 5-6 |
|  |  | D |  |  |  |  |  |  |  |
| LASDB | L22939 | CSRPGNNTRKSVRIGPGQTFYATGDIIGDIRQAHC | 35 | 0 | - | 5 | 2 | 3 | 7 |

^1^Denotes the sample set to which the sequences belong. GB = plasma samples from Guinea-Bissau; Control = control panel; LASDB = sequences from Los Alamos sequence database.

^2^Identification number of the sequence.

^3^Conserved positions are marked with | over the first sequence.

^4^Indicates the charge of amino acids in position 11 and 25.

^5^Number of positively charged amino acids (+), negatively charged amino acids (-), net charge (Net), and total number of charged amino acids (Total).
