# Supplementary material for: Frequent CXCR4 tropism of HIV-1 subtype A and CRF02_AG during late-stage disease - indication of an evolving epidemic in West Africa
Source: Retrovirology. 2010 Mar 22;7:23. doi: 10.1186/1742-4690-7-23 (PMC2855529; doi:10.1186/1742-4690-7-23)
Supplement: Additional file 4 — Table S4 - Alignment and molecular characteristics of HIV-1 CRF02_AG V3 amino acid sequences from study samples and references with determined CXCR4 tropism. Summary of the molecular characteristics of the CXCR4 tropic sequences used in the genotypic analysis. [file 1742-4690-7-23-S4.DOC]

**Additional Table S4 – Alignment and molecular characteristics of HIV-1 CRF02_AG V3 amino acid sequences from study samples and references with determined CXCR4 tropism.**

| **R5X4 and X4** | | | | | | | | | |
| --- | --- | --- | --- | --- | --- | --- | --- | --- | --- |
| **Set^1^** | **Sample^2^** | **V3 sequence^3^** | **Length** | **Position^4^** | | **Charge^5^** | | | |
|  |  | \| \|\| \| \| \| \| \| |  | 11 | 25 | + | - | Net | Total |
| GB | DL1996H | CTRPGNNTRKSVRIGPG--QAFYT-NDIIGDIRQAHC | 34 | 0 | - | 5 | 2 | 3 | 7 |
| GB | DL2089J | CIRPGNNTRKSVRIGPG--QTFYATEGIIGNIRQAYC | 35 | 0 | 0 | 5 | 1 | 4 | 6 |
| GB | DL2249I | CTRPGNNTKKSVRMGPG--RAFYAK-AIIGDIRKAYC | 34 | 0 | 0 | 8-4 | 1-2 | 2-7 | 5-10 |
|  |  | RQ HI Q N E |  |  |  |  |  |  |  |
| GB | DL2339E | CIRPGNNTRKSVRIGPG--QTFFATGDIIGDTRKAHC | 35 | 0 | - | 6 | 2 | 4 | 8 |
| GB | DL2401M | CTRPSNNTRKSVRIGPG--QTFYATGGIIGDIRQAHC | 35 | 0 | 0 | 5 | 1-2 | 3-4 | 6-7 |
|  |  | G D V |  |  |  |  |  |  |  |
| GB | DL2713H | CTRPNNNTRKGIHIGPG--QTFYATGAITGDIRQAHC | 35 | -/0 | -/0 | 3-4 | 1-4 | -1-3 | 4-8 |
|  |  | S I DV A D A |  |  |  |  |  |  |  |
|  |  | D I |  |  |  |  |  |  |  |
|  |  | G V |  |  |  |  |  |  |  |
| GB | DL2920H | CSRPDNNTRKSVRIGPG--QAFYT-MDIIGDIRKAFC | 34 | 0 | - | 6 | 3 | 3 | 9 |
| GB | DL3037E | CTRPSNNTRKGIGIGPG--QTFYATEAIIGDIRQAHC | 35 | 0 | 0 | 4 | 2 | 2 | 6 |
| GB | DL3039G | CTRPNNNTRKSVRIGPG--QTFYAAGDIIGNIRQAHC | 35 | 0 | - | 5 | 1-2 | 3-4 | 6-7 |
|  |  | D |  |  |  |  |  |  |  |
| GB | DL3087E | CIRPGNNTRTSVRLGPG--RTFYATGDIIGDIRQAHC | 35 | 0 | - | 3-5 | 2 | 1-3 | 5-7 |
|  |  | SI Q |  |  |  |  |  |  |  |
| GB | DL3098I | CTRPNNNTRKSVRIGPG--QAFFATGDIIGKIKQAHC | 35 | 0 | - | 5-6 | 1-2 | 3-5 | 6-8 |
|  |  | I V E R |  |  |  |  |  |  |  |
|  |  | N |  |  |  |  |  |  |  |
|  |  | D |  |  |  |  |  |  |  |
| GB | DL3169F | CTRPGNNTRKSIRIGPG--QTFYARGDIIGDIRRAHC | 35 | 0 | - | 6-7 | 2 | 4-5 | 8-9 |
|  |  | S Q |  |  |  |  |  |  |  |
|  |  | I |  |  |  |  |  |  |  |
| GB | DL3170F | CTRPGNNTRKSVRIGPG--QTFYATGDIIGDIRQAHC | 35 | 0 | - | 5 | 2 | 3 | 7 |
|  |  | S E |  |  |  |  |  |  |  |
| GB | DL3234J | CTRPGNNTRKSVRIGPG--QTFYATGDIIGDIRRAHC | 35 | 0 | - | 6 | 2 | 4 | 8 |
|  |  | S |  |  |  |  |  |  |  |
| GB | DL3312E | CTRPGNKRIKSWRIGPG--RTFYA-NGIIGDIRKAHC | 34 | 0 | -/0 | 3-8 | 1-2 | 1-7 | 4-10 |
|  |  | T N N A Q D R Y |  |  |  |  |  |  |  |
|  |  | S H Q |  |  |  |  |  |  |  |
|  |  | S |  |  |  |  |  |  |  |
| GB | DL3633G | CTRPGNNTRKSVRIGPG--QTFYATGDIIGDIRQAYC | 35 | 0 | - | 5 | 2 | 3 | 7 |
|  |  | S |  |  |  |  |  |  |  |
| GB | DL3733G | CTRPNNNTRKSIHIGPG--QAFYATGDIIGDIRQAHC | 35 | 0 | - | 4 | 2 | 2 | 6 |
| GB | DL3721C | CTRPSNNTRKSVRIGPG--QTFYATGEIIGNIRKAYC | 35 | 0 | - | 6 | 1 | 5 | 7 |
| GB | DL4632E | CTRPNNNTRKSIRIGPG--QTFYATGEITGDIRKAYC | 35 | 0 | - | 6 | 2 | 4 | 8 |
| Control | 30405 | CTRPNNNIRKSVRIGIGRGHTFYATGDIIGNIRQAHC | 37 | 0 | - | 6 | 1 | 5 | 7 |
| LASDB | AF355320 | CTRPGNPIRKRIGIGPG--QAFHATGNIIGDIRRAQC | 35 | + | 0 | 6 | 1 | 5 | 7 |
| LASDB | AF355334 | CTRPGNNTRRRMRIGPG--Y-FYTK-RIIGDIRQAHC | 33 | + | + | 8 | 1 | 7 | 9 |
| LASDB | AF355336 | CTRPANNTRRAIGIGPG--RKYYATDKIIGNIRQAHC | 35 | 0 | + | 7 | 1 | 6 | 8 |
| LASDB | AM279356 | CTRPGKIKKRHVRIGPG--RAFWITGDIGGYIRQAHC | 35 | 0 | - | 8 | 1 | 7 | 9 |
| LASDB | AY271690 | CTRPGNPIRKRVGIGPG--QAFHATGNIIGDIRRAQC | 35 | + | 0 | 6 | 1 | 5 | 7 |
| LASDB | DQ825459 | CTRPNNNTRRSLRIGPG--RTFYAAGKIIGDIRQAHC | 35 | 0 | + | 7 | 1 | 6 | 8 |
| LASDB | DQ825460 | CTRPSRITRGRVHIGPG--RAFHATSGITGDIRQAYC | 35 | + | 0 | 6 | 1 | 5 | 7 |
| LASDB | DQ825462 | CTRPNNNTRKGVRIGPG--QTFYATGDIIGNIRQAHC | 35 | 0 | - | 5 | 1 | 4 | 6 |
| LASDB | FJ652334 | CTRPNNNTRKSVRIGPG--QAFYATGDIVGDVRQAHC | 35 | 0 | - | 5 | 2 | 3 | 7 |
| LASDB | FJ652355 | CTRPNNNTIKSIHIGPG--RAFYATGRIVGDIRQAHC | 35 | 0 | +/-/0 | 4-5 | 1-3 | 1-4 | 5-8 |
|  |  | VP DKV |  |  |  |  |  |  |  |
|  |  | G |  |  |  |  |  |  |  |
|  |  | N |  |  |  |  |  |  |  |
|  |  | S |  |  |  |  |  |  |  |
|  |  | D |  |  |  |  |  |  |  |
|  |  | E |  |  |  |  |  |  |  |
| LASDB | FJ652369 | CTRPNNNTRTGIHIGPG--QTFYATGGIIGNIRQAHC | 35 | 0 | 0 | 2-3 | 0-1 | 1-3 | 2-4 |
|  |  | S A D Y |  |  |  |  |  |  |  |
| LASDB | FJ652370 | CTRPSNNTRQSVRIGPG--RTFYATGGIIGDIRQVHC | 35 | 0 | 0 | 3-5 | 1 | 2-4 | 4-6 |
|  |  | S Q T |  |  |  |  |  |  |  |
| LASDB | FJ652373 | CTRPNNNTRRRVHIGPG--RAFYTTGDIIGNIRKAYC | 35 | +/- | - | 5-7 | 1-2 | 3-6 | 6-9 |
|  |  | KS P D Q |  |  |  |  |  |  |  |
|  |  | I |  |  |  |  |  |  |  |
|  |  | L |  |  |  |  |  |  |  |
| LASDB | FJ652374 | CTRPNNNTRESVRIGPG--QTFYAAGRIIGNIRQAHC | 35 | 0 | +/-/0 | 4-5 | 1-3 | 1-4 | 5-8 |
|  |  | K V D |  |  |  |  |  |  |  |
|  |  | G |  |  |  |  |  |  |  |
|  |  | E |  |  |  |  |  |  |  |
| LASDB | FJ652375 | RTRPGNNTIKRIGIGPG--QSWLAHKQKVGDIRQAHC | 35 | + | 0 | 3-7 | 1 | 2-6 | 4-8 |
|  |  | GA VQ Q I |  |  |  |  |  |  |  |
| LASDB | FJ652377 | CTRPNNNTRRSVPIGPG--QTFYARGRIIGDIRRAHC | 35 | 0 | + | 6-7 | 1-3 | 3-6 | 7-10 |
|  |  | YSD G D Q |  |  |  |  |  |  |  |

^1^Denotes the sample set to which the sequences belong. GB = plasma samples from Guinea-Bissau; Control = control panel; LASDB = sequences from Los Alamos sequence database.

^2^Identification number of the sequence.

^3^Conserved positions are marked with | over the first sequence.

^4^Indicates the charge of amino acids in position 11 and 25.

^5^Number of positively charged amino acids (+), negatively charged amino acids (-), net charge (Net), and total number of charged amino acids (Total).
