# Supplementary material for: Frequent CXCR4 tropism of HIV-1 subtype A and CRF02_AG during late-stage disease - indication of an evolving epidemic in West Africa
Source: Retrovirology. 2010 Mar 22;7:23. doi: 10.1186/1742-4690-7-23 (PMC2855529; doi:10.1186/1742-4690-7-23)
Supplement: Additional file 5 — Table S5. Data used to investigate an evolving epidemic available data from HIV-1 CRF02_AG. Summary of the data obtained from the literature and Los Alamos Sequence Data Base to investigate if the HIV-1 CRF02_AG epidemic represents an evolving epidemic. [file 1742-4690-7-23-S5.DOC]

**Additional Table S5. Data used to investigate an evolving epidemic available data from HIV-1 CRF02_AG.**

| **Country** | **1997-2001** | | **2003-2007** | | **Reference** |
| --- | --- | --- | --- | --- | --- |
|  | **R5/NSI** | **X4/SI** | **R5/NSI** | **X4/SI** |  |
| Cameroon | 19 | 23 | - | - | [1] |
| Ghana | - | - | 1 | - | [2] |
| Guinea-Bissau | 3 | 5 | - | 14 | Data obtained in this study |

**References**

1. Vergne L, Bourgeois A, Mpoudi-Ngole E, Mougnutou R, Mbuagbaw J, Liegeois F, Laurent C, Butel C, Zekeng L, Delaporte E, Peeters M: **Biological and genetic characteristics of HIV infections in Cameroon reveals dual group M and O infections and a correlation between SI-inducing phenotype of the predominant CRF02_AG variant and disease stage.** *Virology* 2003, **310:**254-266.

2. Brandful JA, Coetzer ME, Cilliers T, Phoswa M, Papathanasopoulos MA, Morris L, Moore PL: **Phenotypic characterization of HIV type 1 isolates from Ghana.** *AIDS Res Hum Retroviruses* 2007, **23:**144-152.
