# Supplementary material for: Frequent CXCR4 tropism of HIV-1 subtype A and CRF02_AG during late-stage disease - indication of an evolving epidemic in West Africa
Source: Retrovirology. 2010 Mar 22;7:23. doi: 10.1186/1742-4690-7-23 (PMC2855529; doi:10.1186/1742-4690-7-23)
Supplement: Additional file 6 — Table S6 - Overview of the literature analysis. Summary of the data obtained from the literature and used to determine the amount of CXCR4 tropism in late stage disease in the studied HIV-1 subtypes. [file 1742-4690-7-23-S6.DOC]

**Additional Table S6. Overview of the literature analysis.**

| **Subtype** | **Number of subjects^1^** | | | **References^2^** |
| --- | --- | --- | --- | --- |
|  | **R5/NSI** | **X4/SI** | **Total** |  |
| A | 18 | 27 | 45 | [1-6], data obtained in this study |
| B | 40 | 80 | 120 | [2, 3, 7-15] |
| C | 176 | 32 | 208 | [2, 4, 5, 16-25] |
| D | 11 | 17 | 28 | [1, 2, 4, 5] |
| CRF01_AE | 6 | 20 | 26 | [2, 4, 26] |
| CRF02_AG | 25 | 46 | 71 | [5, 6, 27], data obtained in this study |

^1^Number of subjects included. Only subjects in late-stage disease (diagnosed with AIDS or CD4 T cell count ≤200 cell/µl) were included, and in cases were the same patient appeared in several studies the patient data were only used once.

^2^References from were the data were collected. For references [2, 4, 7-14] subtype and/or coreceptor tropism were verified by personal communication with the authors.

**References**

1. Kaleebu P, Nankya IL, Yirrell DL, Shafer LA, Kyosiimire-Lugemwa J, Lule DB, Morgan D, Beddows S, Weber J, Whitworth JA: **Relation between chemokine receptor use, disease stage, and HIV-1 subtypes A and D: results from a rural Ugandan cohort.** *J Acquir Immune Defic Syndr* 2007, **45:**28-33.

2. Tscherning C, Alaeus A, Fredriksson R, Bjorndal A, Deng H, Littman DR, Fenyo EM, Albert J: **Differences in chemokine coreceptor usage between genetic subtypes of HIV-1.** *Virology* 1998, **241:**181-188.

3. Peeters M, Vincent R, Perret JL, Lasky M, Patrel D, Liegeois F, Courgnaud V, Seng R, Matton T, Molinier S, Delaporte E: **Evidence for differences in MT2 cell tropism according to genetic subtypes of HIV-1: syncytium-inducing variants seem rare among subtype C HIV-1 viruses.** *J Acquir Immune Defic Syndr Hum Retrovirol* 1999, **20:**115-121.

4. Bjorndal A, Sonnerborg A, Tscherning C, Albert J, Fenyo EM: **Phenotypic characteristics of human immunodeficiency virus type 1 subtype C isolates of Ethiopian AIDS patients.** *AIDS Res Hum Retroviruses* 1999, **15:**647-653.

5. Vergne L, Bourgeois A, Mpoudi-Ngole E, Mougnutou R, Mbuagbaw J, Liegeois F, Laurent C, Butel C, Zekeng L, Delaporte E, Peeters M: **Biological and genetic characteristics of HIV infections in Cameroon reveals dual group M and O infections and a correlation between SI-inducing phenotype of the predominant CRF02_AG variant and disease stage.** *Virology* 2003, **310:**254-266.

6. Brandful JA, Coetzer ME, Cilliers T, Phoswa M, Papathanasopoulos MA, Morris L, Moore PL: **Phenotypic characterization of HIV type 1 isolates from Ghana.** *AIDS Res Hum Retroviruses* 2007, **23:**144-152.

7. Tersmette M, de Goede RE, Al BJ, Winkel IN, Gruters RA, Cuypers HT, Huisman HG, Miedema F: **Differential syncytium-inducing capacity of human immunodeficiency virus isolates: frequent detection of syncytium-inducing isolates in patients with acquired immunodeficiency syndrome (AIDS) and AIDS-related complex.** *J Virol* 1988, **62:**2026-2032.

8. Tersmette M, Lange JM, de Goede RE, de Wolf F, Eeftink-Schattenkerk JK, Schellekens PT, Coutinho RA, Huisman JG, Goudsmit J, Miedema F: **Association between biological properties of human immunodeficiency virus variants and risk for AIDS and AIDS mortality.** *Lancet* 1989, **1:**983-985.

9. Schuitemaker H, Kootstra NA, de Goede RE, de Wolf F, Miedema F, Tersmette M: **Monocytotropic human immunodeficiency virus type 1 (HIV-1) variants detectable in all stages of HIV-1 infection lack T-cell line tropism and syncytium-inducing ability in primary T-cell culture.** *J Virol* 1991, **65:**356-363.

10. Koot M, Vos AH, Keet RP, de Goede RE, Dercksen MW, Terpstra FG, Coutinho RA, Miedema F, Tersmette M: **HIV-1 biological phenotype in long-term infected individuals evaluated with an MT-2 cocultivation assay.** *Aids* 1992, **6:**49-54.

11. Schuitemaker H, Koot M, Kootstra NA, Dercksen MW, de Goede RE, van Steenwijk RP, Lange JM, Schattenkerk JK, Miedema F, Tersmette M: **Biological phenotype of human immunodeficiency virus type 1 clones at different stages of infection: progression of disease is associated with a shift from monocytotropic to T-cell-tropic virus population.** *J Virol* 1992, **66:**1354-1360.

12. Karlsson A, Parsmyr K, Sandstrom E, Fenyo EM, Albert J: **MT-2 cell tropism as prognostic marker for disease progression in human immunodeficiency virus type 1 infection.** *J Clin Microbiol* 1994, **32:**364-370.

13. Bjorndal A, Deng H, Jansson M, Fiore JR, Colognesi C, Karlsson A, Albert J, Scarlatti G, Littman DR, Fenyo EM: **Coreceptor usage of primary human immunodeficiency virus type 1 isolates varies according to biological phenotype.** *J Virol* 1997, **71:**7478-7487.

14. Scarlatti G, Tresoldi E, Bjorndal A, Fredriksson R, Colognesi C, Deng HK, Malnati MS, Plebani A, Siccardi AG, Littman DR, et al: **In vivo evolution of HIV-1 co-receptor usage and sensitivity to chemokine-mediated suppression.** *Nat Med* 1997, **3:**1259-1265.

15. Boucher CA, Lange JM, Miedema FF, Weverling GJ, Koot M, Mulder JW, Goudsmit J, Kellam P, Larder BA, Tersmette M: **HIV-1 biological phenotype and the development of zidovudine resistance in relation to disease progression in asymptomatic individuals during treatment.** *Aids* 1992, **6:**1259-1264.

16. Abebe A, Demissie D, Goudsmit J, Brouwer M, Kuiken CL, Pollakis G, Schuitemaker H, Fontanet AL, Rinke de Wit TF: **HIV-1 subtype C syncytium- and non-syncytium-inducing phenotypes and coreceptor usage among Ethiopian patients with AIDS.** *Aids* 1999, **13:**1305-1311.

17. Ping LH, Nelson JA, Hoffman IF, Schock J, Lamers SL, Goodman M, Vernazza P, Kazembe P, Maida M, Zimba D, et al: **Characterization of V3 sequence heterogeneity in subtype C human immunodeficiency virus type 1 isolates from Malawi: underrepresentation of X4 variants.** *J Virol* 1999, **73:**6271-6281.

18. Cilliers T, Nhlapo J, Coetzer M, Orlovic D, Ketas T, Olson WC, Moore JP, Trkola A, Morris L: **The CCR5 and CXCR4 coreceptors are both used by human immunodeficiency virus type 1 primary isolates from subtype C.** *J Virol* 2003, **77:**4449-4456.

19. Ndung'u T, Sepako E, McLane MF, Chand F, Bedi K, Gaseitsiwe S, Doualla-Bell F, Peter T, Thior I, Moyo SM, et al: **HIV-1 subtype C in vitro growth and coreceptor utilization.** *Virology* 2006, **347:**247-260.

20. Coetzer M, Cilliers T, Ping LH, Swanstrom R, Morris L: **Genetic characteristics of the V3 region associated with CXCR4 usage in HIV-1 subtype C isolates.** *Virology* 2006, **356:**95-105.

21. Connell BJ, Michler K, Capovilla A, Venter WD, Stevens WS, Papathanasopoulos MA: **Emergence of X4 usage among HIV-1 subtype C: evidence for an evolving epidemic in South Africa.** *Aids* 2008, **22:**896-899.

22. Choge I, Cilliers T, Walker P, Taylor N, Phoswa M, Meyers T, Viljoen J, Violari A, Gray G, Moore PL, et al: **Genotypic and phenotypic characterization of viral isolates from HIV-1 subtype C-infected children with slow and rapid disease progression.** *AIDS Res Hum Retroviruses* 2006, **22:**458-465.

23. Cecilia D, Kulkarni SS, Tripathy SP, Gangakhedkar RR, Paranjape RS, Gadkari DA: **Absence of coreceptor switch with disease progression in human immunodeficiency virus infections in India.** *Virology* 2000, **271:**253-258.

24. Johnston ER, Zijenah LS, Mutetwa S, Kantor R, Kittinunvorakoon C, Katzenstein DA: **High frequency of syncytium-inducing and CXCR4-tropic viruses among human immunodeficiency virus type 1 subtype C-infected patients receiving antiretroviral treatment.** *J Virol* 2003, **77:**7682-7688.

25. Morris L, Cilliers T, Bredell H, Phoswa M, Martin DJ: **CCR5 is the major coreceptor used by HIV-1 subtype C isolates from patients with active tuberculosis.** *AIDS Res Hum Retroviruses* 2001, **17:**697-701.

26. Yu XF, Wang Z, Beyrer C, Celentano DD, Khamboonruang C, Allen E, Nelson K: **Phenotypic and genotypic characteristics of human immunodeficiency virus type 1 from patients with AIDS in northern Thailand.** *J Virol* 1995, **69:**4649-4655.

27. Bikandou B, Takehisa J, Mboudjeka I, Ido E, Kuwata T, Miyazaki Y, Moriyama H, Harada Y, Taniguchi Y, Ichimura H, et al: **Genetic subtypes of HIV type 1 in Republic of Congo.** *AIDS Res Hum Retroviruses* 2000, **16:**613-619.
