# Supplementary material for: Frequent CXCR4 tropism of HIV-1 subtype A and CRF02_AG during late-stage disease - indication of an evolving epidemic in West Africa
Source: Retrovirology. 2010 Mar 22;7:23. doi: 10.1186/1742-4690-7-23 (PMC2855529; doi:10.1186/1742-4690-7-23)
Supplement: Additional file 7 — Table S7 - Overview of the subtype C material. Summary of the subtype C data obtained from the literature review used in the analysis of an evolving epidemic for subtype C. [file 1742-4690-7-23-S7.DOC]

**Additional Table S7. Overview of the subtype C material**.

| **Country** | **Sampling years** | **Number of subjects** | **References** |
| --- | --- | --- | --- |
| Cameroon | before 2000 | 1 | [1] |
| Ethiopia | 1987-1999 | 57 | [2, 3] |
| India | before 1999 | 29 | [4] |
| Malawi | 1996 | 8 | [5] |
| South Africa | 1995-2005 | 81 | [6-11] |
| Sweden | before 1998 | 4 | [12] |
| Zimbabwe | 2001 | 28 | [13] |

^1^Number of subjects included and references from were the data were collected. Only subjects in late-stage disease (diagnosed with AIDS or CD4 T cell count ≤200 cell/µl) were included, and in cases were the same patient appeared in several studies the patient data were only used once.

^2^References from were the data were collected.

**References**

1. Vergne L, Bourgeois A, Mpoudi-Ngole E, Mougnutou R, Mbuagbaw J, Liegeois F, Laurent C, Butel C, Zekeng L, Delaporte E, Peeters M: **Biological and genetic characteristics of HIV infections in Cameroon reveals dual group M and O infections and a correlation between SI-inducing phenotype of the predominant CRF02_AG variant and disease stage.** *Virology* 2003, **310:**254-266.

2. Abebe A, Demissie D, Goudsmit J, Brouwer M, Kuiken CL, Pollakis G, Schuitemaker H, Fontanet AL, Rinke de Wit TF: **HIV-1 subtype C syncytium- and non-syncytium-inducing phenotypes and coreceptor usage among Ethiopian patients with AIDS.** *Aids* 1999, **13:**1305-1311.

3. Bjorndal A, Sonnerborg A, Tscherning C, Albert J, Fenyo EM: **Phenotypic characteristics of human immunodeficiency virus type 1 subtype C isolates of Ethiopian AIDS patients.** *AIDS Res Hum Retroviruses* 1999, **15:**647-653.

4. Cecilia D, Kulkarni SS, Tripathy SP, Gangakhedkar RR, Paranjape RS, Gadkari DA: **Absence of coreceptor switch with disease progression in human immunodeficiency virus infections in India.** *Virology* 2000, **271:**253-258.

5. Ping LH, Nelson JA, Hoffman IF, Schock J, Lamers SL, Goodman M, Vernazza P, Kazembe P, Maida M, Zimba D, et al: **Characterization of V3 sequence heterogeneity in subtype C human immunodeficiency virus type 1 isolates from Malawi: underrepresentation of X4 variants.** *J Virol* 1999, **73:**6271-6281.

6. Morris L, Cilliers T, Bredell H, Phoswa M, Martin DJ: **CCR5 is the major coreceptor used by HIV-1 subtype C isolates from patients with active tuberculosis.** *AIDS Res Hum Retroviruses* 2001, **17:**697-701.

7. Cilliers T, Nhlapo J, Coetzer M, Orlovic D, Ketas T, Olson WC, Moore JP, Trkola A, Morris L: **The CCR5 and CXCR4 coreceptors are both used by human immunodeficiency virus type 1 primary isolates from subtype C.** *J Virol* 2003, **77:**4449-4456.

8. Coetzer M, Cilliers T, Ping LH, Swanstrom R, Morris L: **Genetic characteristics of the V3 region associated with CXCR4 usage in HIV-1 subtype C isolates.** *Virology* 2006, **356:**95-105.

9. Ndung'u T, Sepako E, McLane MF, Chand F, Bedi K, Gaseitsiwe S, Doualla-Bell F, Peter T, Thior I, Moyo SM, et al: **HIV-1 subtype C in vitro growth and coreceptor utilization.** *Virology* 2006, **347:**247-260.

10. Connell BJ, Michler K, Capovilla A, Venter WD, Stevens WS, Papathanasopoulos MA: **Emergence of X4 usage among HIV-1 subtype C: evidence for an evolving epidemic in South Africa.** *Aids* 2008, **22:**896-899.

11. Choge I, Cilliers T, Walker P, Taylor N, Phoswa M, Meyers T, Viljoen J, Violari A, Gray G, Moore PL, et al: **Genotypic and phenotypic characterization of viral isolates from HIV-1 subtype C-infected children with slow and rapid disease progression.** *AIDS Res Hum Retroviruses* 2006, **22:**458-465.

12. Tscherning C, Alaeus A, Fredriksson R, Bjorndal A, Deng H, Littman DR, Fenyo EM, Albert J: **Differences in chemokine coreceptor usage between genetic subtypes of HIV-1.** *Virology* 1998, **241:**181-188.

13. Johnston ER, Zijenah LS, Mutetwa S, Kantor R, Kittinunvorakoon C, Katzenstein DA: **High frequency of syncytium-inducing and CXCR4-tropic viruses among human immunodeficiency virus type 1 subtype C-infected patients receiving antiretroviral treatment.** *J Virol* 2003, **77:**7682-7688.
